# Hybrid Materials Based on Poly-3-amine-7-methylamine-2-methylphenazine and Magnetite Nanoparticles Immobilized on Single-Walled Carbon Nanotubes

**DOI:** 10.3390/polym10050544

**Published:** 2018-05-18

**Authors:** Sveta Zhiraslanovna Ozkan, Galina Petrovna Karpacheva, Petr Aleksandrovich Chernavskii, Ella Leont’evna Dzidziguri, Galina Nikolaevna Bondarenko, Galina Viktorovna Pankina

**Affiliations:** 1 A.V. Topchiev Institute of Petrochemical Synthesis, Russian Academy of Sciences, 29 Leninsky Prospect, Moscow 119991, Russia; gpk@ips.ac.ru (G.P.K.); bond@ips.ac.ru (G.N.B.); 2 Department of Chemistry, Lomonosov Moscow State University, 1-3 Leninskie Gory, Moscow 119991, Russia; chern5@inbox.ru (P.A.C.); pankina5151@inbox.ru (G.V.P.); 3 Department of Functional Nanosystems and High-Temperature Materials, National University of Science and Technology MISIS, Leninsky Prospect, Moscow 119049, Russia; avrore@gmail.com

**Keywords:** poly-3-amine-7-methylamine-2-methylphenazine, conjugated polymers, in situ oxidative polymerization, polymer-metal-carbon nanocomposite, single-walled carbon nanotubes, magnetite nanoparticles

## Abstract

Polymer-metal-carbon hybrid nanomaterials based on thermostable electroactive poly-3-amine-7-methylamine-2-methylphenazine (PAMMP), single walled carbon nanotubes (SWCNT), and magnetite (Fe_3_O_4_) nanoparticles were synthesized for the first time. Hybrid Fe_3_O_4_/SWCNT/PAMMP nanomaterial synthesis was carried out via in situ chemical oxidative polymerization of 3-amine-7-methylamine-2-methylphenazine hydrochloride in the presence of metal-carbon Fe_3_O_4_/SWCNT nanocomposites. Fe_3_O_4_/SWCNT nanocomposites were obtained by the immobilization of magnetite nanoparticles on the SWCNT surface in the course of Fe_3_O_4_ nanoparticles synthesis in alkaline medium. The developed nanocomposite materials were characterized by FTIR spectroscopy, X-ray diffraction (XRD), transmission electron microscopy (TEM), field emission (FE-SEM) scanning electron microscopy, atomic absorption spectrometry (AAS), differential scanning calorimetry (DSC), thermogravimetric analysis (TGA), and magnetometry. The chemical structure and phase composition, magnetic and electrical properties, and thermal stability of the obtained multifunctional nanomaterials, depending on synthesis conditions, were investigated.

## 1. Introduction

Modern technology requires the creation of new generation materials with improved functional characteristics. Hybrid nanomaterials, described here, have a combination of organic and inorganic components providing a range of required properties [1,2,3]. In this respect, hybrid nanomaterials, in which the organic component is a conjugated polymer and the inorganic component is represented by magnetic nanoparticles, are of particular interest to researchers around the globe [4]. Hybrid nanomaterials based on polyconjugated systems and magnetic nanoparticles appear to be very promising for modern technologies due to the combination of their electrical and magnetic properties. Such electromagnetic nanocomposites can be used as cathode materials for chemical batteries [5,6], as anticorrosive coatings [7,8,9,10] and heterogeneous catalyst systems [11], for medical purposes [12], as well as effective sorbents for the purification of drinking water [13,14,15,16,17,18], and as materials that efficiently absorb electromagnetic radiation [19,20,21,22,23]. Therefore, the design of novel nanocomposite materials based on polymers with a system of polyconjugation and magnetic nanoparticles have not lost their relevance. In recent years, much attention has been attracted by new ternary hybrid nanocomposites, which contain, along with conjugated polymers and magnetic nanoparticles, carbon nanomaterials. However, there are only a few works describing the synthesis and properties of ternary nanomaterials containing conjugated polymers, magnetic nanoparticles, and carbon components.

There are two approaches for preparing ternary nanocomposites: in situ oxidative polymerization of aniline in the presence of multiwalled carbon nanotubes (MWCNT), or graphene oxide followed by the deposition of magnetite nanoparticles on their surface [19,20,24]; the in situ oxidative polymerization of aniline or pyrrole in the presence of magnetite nanoparticles anchored to MWCNT or reduced graphene oxide [21,22]. At high dispersity of magnetic nanoparticles the ternary nanocomposites are superparamagnetic [19,20,21]. The saturation magnetization value depends on the content of the magnetic nanoparticles. The aggregation of Fe_3_O_4_ nanoparticles determines the ferromagnetic behavior of the nanocomposite [22]. The obtained nanomaterials can effectively absorb electromagnetic radiation.

**Figure 1:**
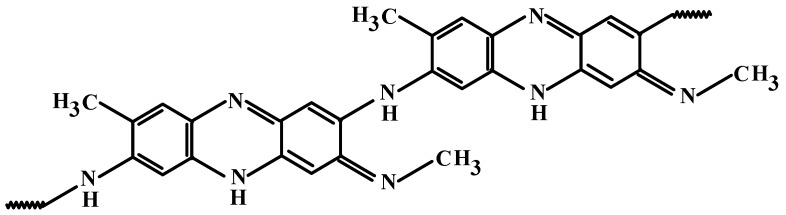
Chemical structure of poly-3-amine-7-methylamine-2-methylphenazine (PAMMP).

In the present paper, a synthesis method is proposed for novel hybrid polymer-metal-carbon nanocomposite materials based on Fe_3_O_4_ nanoparticles immobilized on the surface of single-walled carbon nanotubes (SWCNT). The polymer component of the hybrid nanomaterial is represented by poly-3-amine-7-methylamine-2-methylphenazine (PAMMP), which was synthesized by the authors for the first time via oxidative polymerization of 3-amine-7-dimethylamine-2-methylphenazine hydrochloride (ADMPC)—also known as Neutral Red [25]. PAMMP is a semi-ladder heterocyclic thermostable polymer, in which nitrogen atoms participate in the common polyconjugated system. Analysis of the results of spectral studies by FTIR and electron spectroscopy, X-ray photoelectron spectroscopy, solid state HRMAS (high-resolution magic angle spinning), and ^13^C NMR spectroscopy [25], allow for the representation of a chemical structure of PAMMP, as shown in Figure 1. In the course of the synthesis, PAMMP can form film coatings on the surface of the substrate added to the reaction solution.

**Figure 2:**
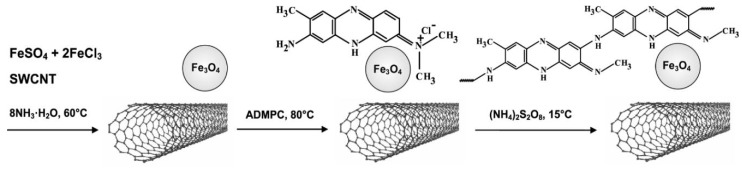
Synthesis scheme of the polymer-metal-carbon hybrid nanomaterial based on poly-3-amine-7-methylamine-2-methylphenazine and Fe_3_O_4_ nanoparticles immobilized on single-walled carbon nanotubes.

The synthesis of hybrid Fe_3_O_4_/SWCNT/PAMMP nanomaterials was carried out in aqueous solutions of acetonitrile or DMF via in situ chemical oxidative polymerization of ADMPC on the surface of the previously synthesized metal-carbon Fe_3_O_4_/SWCNT nanocomposite. The formation methods of the hybrid dispersed Fe_3_O_4_/SWCNT/PAMMP nanomaterial include monomer immobilization on the surface of the metal-carbon Fe_3_O_4_/SWCNT nanocomposite and subsequent in situ oxidative polymerization in a neutral medium in the presence of ammonium persulfate as an oxidant (Figure 2). The magnetic, thermal, and electrical properties of obtained nanomaterials were studied.

## 2. Experimental

Ammonium persulfate (analytical grade) was purified by recrystallization from distilled water as previously described [26]. 3-amine-7-dimethylamine-2-methylphenazine hydrochloride (C_15_H_17_CIN_4_) (ADMPC) (Neutral Red), aqueous ammonia (reagent grade), FeCl_3_·6H_2_O (high-purity grade), and FeSO_4_·7H_2_O (high-purity grade), as well as acetonitrile and DMF (all from Acros Organics, Geel, Belgium), were used without any additional purification. The aqueous solutions of reagents were prepared with distilled water. SWCNT from Carbon Chg, Ltd. (Moscow, Russia) were produced using the electric arc discharge technique with a Ni/Y catalyst (*d* = 1.4–1.6 nm, *l* = 0.5–1.5 µm). PAMMP was obtained via oxidative polymerization in an aqueous solution of acetonitrile [25].

The synthesis of Fe_3_O_4_ nanoparticles immobilized on the SWCNT surface (metal-carbon Fe_3_O_4_/SWCNT nanocomposite) was performed via hydrolysis of iron (II) and (III) salts taken at a ratio 1:2 in a solution of ammonium hydroxide [27] in the presence of SWCNT at 60 °C. For this purpose 0.86 g of FeSO_4_·7H_2_O and 2.35 g of FeCl_3_·6H_2_O were dissolved in 20 mL of distilled water. 0.038 g SWCNT (10 wt % from the weight of ADMPC) was added to the reaction media which was then heated to 60 °C; 5 mL of NH_4_OH was added subsequently. The resulting suspension was heated on a water bath to 80 °C and stirred for 0.5 h. The suspension was cooled at room temperature with continuous intense stirring for 1 h. The obtained Fe_3_O_4_/SWCNT nanocomposite was filtered off, washed with distilled water to remove residual amounts of ammonium hydroxide until the pH value of the filtrate reached 7, and dried over KOH under vacuum to constant weight. The yield of Fe_3_O_4_/SWCNT was 1.072 g with *C*_Fe_ = 61.2% (according to AAS data).

The synthesis of the Fe_3_O_4_/SWCNT/PAMMP nanocomposite was conducted in a neutral medium as follows. Freshly prepared Fe_3_O_4_/SWCNT nanocomposite in the desired concentration (Table 1) was washed with distilled water up to neutral reaction and without pre-drying, was added straight into the ADMPC solution in acetonitrile (0.02 mol/L, 0.38 g). The content of carbon nanotubes *C*_SWCNT_ = 10 and 20 wt % relative to the monomer weight. This process of monomer immobilization on the Fe_3_O_4_/SWCNT surface was carried out at 60 °C with continuous intense stirring for 1 h. The suspension was cooled at room temperature with intense stirring for 1 h. After that, in order to perform in situ oxidative polymerization of ADMPC on the metal-carbon Fe_3_O_4_/SWCNT surface, an aqueous solution (30 mL) of ammonium persulphate (0.04 mol/L, 0.548 g) was added dropwise to the Fe_3_O_4_/SWCNT/ADMPC suspension in acetonitrile, pre-cooled to 15 °C by using the LOIP FT-311-25 cryothermostat (Saint-Petersburg, Russia). The volume ratio of organic and aqueous phases was 1:1 (*V*_total_ = 60 mL). The synthesis continued for 4 h with intense stirring at 15 °C. When the synthesis was completed, the mixture was precipitated in a fivefold excess of distilled water. The resulting product was filtered off, washed repeatedly with distilled water to remove residual amounts of reagent, and dried over KOH under vacuum to constant weight. Oxidative polymerization of ADMPC in the presence of the Fe_3_O_4_/SWCNT nanocomposite can also be carried out in an aqueous solution of DMF. The yield of Fe_3_O_4_/SWCNT/PAMMP was 1.23 g at *C*_Fe_ = 45.7% (according to AAS data) (Table 1).

In order to synthesize the SWCNT/PAMMP composite material, ADMPC (0.02 mol/L, 0.38 g) was dissolved in acetonitrile (30 mL). 0.0114 g SWCNT (3 wt % relative to the monomer weight) was added to the resulting solution. The process was carried out at 60 °C with continuous intense stirring for 1 h. The resulting SWCNT/ADMPC suspension was stirred in an ultrasonic bath at room temperature for 0.5 h. Then, to perform in situ oxidative polymerization of ADMPC in the presence of SWCNT, an aqueous solution (30 mL) of ammonium persulphate (0.04 mol/L, 0.548 g) was added dropwise to the SWCNT/ADMPC suspension, pre-cooled to 15 °C. The volume ratio of organic and aqueous phases was 1:1 (*V*_total_ = 60 mL). The synthesis continued for 4 h with intense stirring at 15 °C. When the synthesis was completed, the mixture was precipitated in a fivefold excess of distilled water. The resulting product was filtered off, washed repeatedly with distilled water to remove residual amounts of reagent, and dried over KOH under vacuum to constant weight. The yield of SWCNT/PAMMP was 0.23 g.

The content of metal in Fe_3_O_4_/SWCNT and Fe_3_O_4_/SWCNT/PAMMP nanocomposite materials was measured quantitatively by atomic absorption spectrometry using a Carl Zeiss JENA AAS 30 spectrophotometer (Schwerin, Germany, Table 1). Fe content was measured to ±1% accuracy.

FTIR spectra of the samples were measured on a Bruker IFS 66v FTIR spectrometer (Karlsruhe, Germany) in the range of 400–4000 cm^−1^. The samples were prepared as KBr pressed pellets. Attenuated total reflection (ATR) FTIR spectra in the attenuated total reflectance mode were recorded using a HYPERION-2000 IR microscope (Bruker, Karlsruhe, Germany) and coupled with the Bruker IFS 66v FTIR spectrometer in the range of 600–4000 cm^−1^ (150 scans, ZnSe crystal, resolution of 2 cm^−1^).

X-ray diffraction study was performed in ambient atmosphere using a Difray-401 X-ray diffractometer with Bragg–Bretano (Scientific Instruments Joint Stock Company, Saint-Petersburg, Russia) focusing on Cr*K*_α_ radiation, λ = 0.229 nm. The results of X-ray diffraction analysis were used to calculate the size distribution of the coherent scattering regions of crystallites [28] in Fe_3_O_4_ nanoparticles.

An electron microscopic study was performed using a LEO 912 AB OMEGA transmission electron microscope (Bioz Inc., Los Altos, CA, USA) and a Zeiss Supra 25 FE-SEM field emission scanning electron microscope (Carl Zeiss AG, Jena, Germany). To prepare the TEM samples, the nanocomposites were thoroughly ground in an agate mortar and the dispersed powder was applied to a supporting grid. The resolution of the resulting images is 1–2 nm. The size of nanoparticles is determined using the EsiVision software (eVision Software, The Hague, The Netherlands).

The BET surface area test was conducted on Micromeritics ASAP 2020 (Micromeritics Corporate, Norcross, GA, USA) via capillary nitrogen condensation method at 77 K in the region of relative pressure (P/P_0_) from 0.01 to 0.99. The surface area correction factor was ±1. Degassing of the sample was carried out at 120 °C for 2 h.

A vibration magnetometer was used to study the magnetic characteristics of the systems. The cell of the vibration magnetometer was designed as a flow quartz microreactor, which made it possible to study chemical transformations in “in situ mode” [29]. Specific magnetization depending on the magnetic field value was measured; magnetic characteristics of the samples at room temperature were determined.

The DC conductivity at room temperature was determined by a standard 4-point procedure with a Loresta-GP MCP-T610 unit (Mitsubishi, Shimotsuruma, Japan). The AC conductivity was measured with a 6367A precision LCR-meter (Microtest, New Taipei City, Taiwan) in the frequency range of 0.1 Hz–1.15 MHz.

Thermogravimetric analysis (TGA) was performed on a Mettler Toledo TGA/DSC1 (Giessen, Germany) in the dynamic mode in the range of 30–1000 °C in air and in the argon flow. The weight of the samples was 100 mg, the heating rate was 10 °C/min, and the argon flow velocity was 10 mL/min. Calcined aluminum oxide was used as a reference. The samples were analyzed in an Al_2_O_3_ crucible.

Differential scanning calorimetry (DSC) was performed on a Mettler Toledo DSC823^ee^ calorimeter (Giessen, Germany). The samples were heated at the rate of 10 °C/min in the nitrogen atmosphere, with a nitrogen flow rate of 70 mL/min. The measurement results were processed with the service program STARe supplied with the device.

## 3. Results and Discussion

The formation of Fe_3_O_4_/SWCNT and Fe_3_O_4_/SWCNT/PAMMP nanocomposite materials was confirmed by transmission (TEM) and scanning (FE-SEM) electron microscopy, FTIR spectroscopy, X-ray diffraction, and atomic absorption spectrometry (AAS).

**Figure 3:**
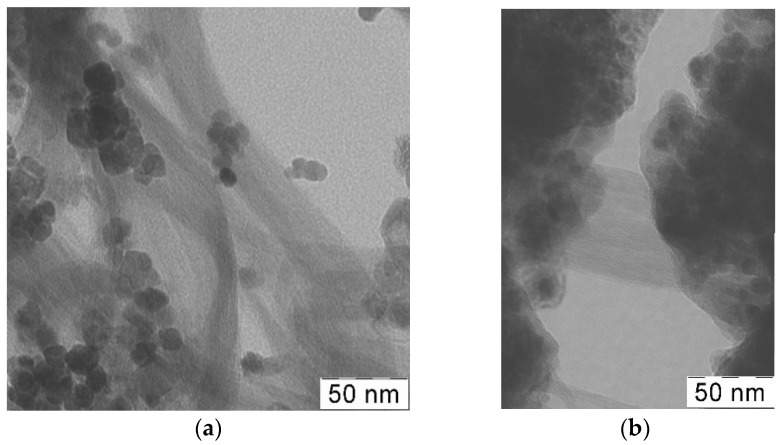
TEM images of Fe_3_O_4_/SWCNT (**a**) and Fe_3_O_4_/SWCNT/PAMMP (**b**) nanocomposites.

The synthesis method of the metal-carbon Fe_3_O_4_/SWCNT nanocomposite via precipitation of magnetite nanoparticles on the surface of SWCNT is original: Due to the presence of SWCNT in the reaction medium, when the mixture of iron (II) and (III) salts hydrolyzes in a solution of ammonium hydroxide, the formation of Fe_3_O_4_ nanoparticles occurs, and they are simultaneously anchored onto the SWCNT surface (Figure 3a).

**Figure 4:**
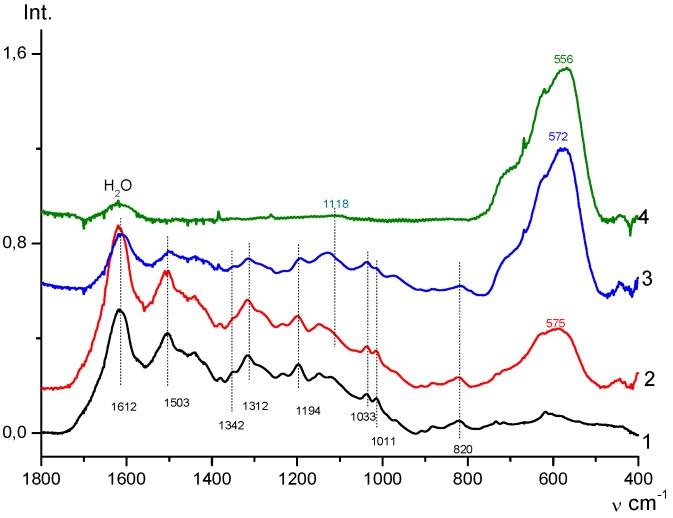
FTIR spectra of PAMMP (1), Fe_3_O_4_/SWCNT/PAMMP (2,3), and Fe_3_O_4_/SWCNT (4) nanocomposites, prepared at *C*_Fe_ = 17.6 (2), 45.7 (3) and 61.2% (4).

Thus, the immobilization of magnetite nanoparticles on the SWCNT surface was carried out directly in the alkaline medium where the Fe_3_O_4_ nanoparticles were prepared. The FTIR spectrum of Fe_3_O_4_/SWCNT nanocomposite (Figure 4) shows a band at 556 cm^−1^ alongside a band at 430 cm^−1^, characterizing the stretching vibrations of the magnetite ν_Fe–O_ bond.

**Figure 5:**
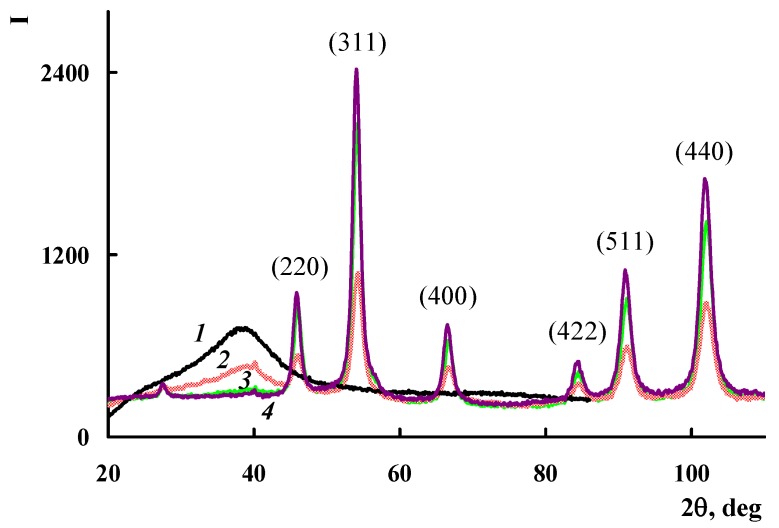
X-ray diffractograms of PAMMP (1), Fe_3_O_4_/SWCNT/PAMMP (2,3), and Fe_3_O_4_/SWCNT (4) nanocomposites, prepared at *C*_Fe_ = 17.6 (2), 45.7 (3) and 61.2% (4).

The X-ray diffraction analysis of the metal-carbon Fe_3_O_4_/SWCNT nanocomposite structure made it is possible to establish that the only metal-containing phase of the nanocomposite is the Fe_3_O_4_ phase, identified clearly by diffraction peaks at scattering angles 2θ = 45.97°, 54.1°, 66.69°, 84.57°, 90.97°, and 102.16° (Cr*K*_α_-radiation) (Figure 5) [30,31]. All these diffraction peaks correspond to the cubic structure of Fe_3_O_4_ (JCPDS 19-0629) and refer to the Miller Indices (220), (311), (400), (422), (511), and (440) [32]. The absence of the carbon phase diffraction peak on the diffractogram of the Fe_3_O_4_/SWCNT nanocomposite was explained by the impossibility of obtaining a diffraction pattern from a single SWCNT plane.

Based on the obtained metal-carbon Fe_3_O_4_/SWCNT nanocomposite, polymer-metal-carbon hybrid Fe_3_O_4_/SWCNT/PAMMP nanomaterials were synthesized via in situ oxidative polymerization of ADMPC in an aqueous solution of acetonitrile. To immobilize ADMPC on the Fe_3_O_4_/SWCNT nanocomposite surface, freshly prepared magnetite nanoparticles precipitated to SWCNT and were washed until neutral reaction without pre-drying, then were added to the reaction neutral medium of Fe_3_O_4_/SWCNT/PAMMP nanomaterial synthesis.

**Figure 6:**
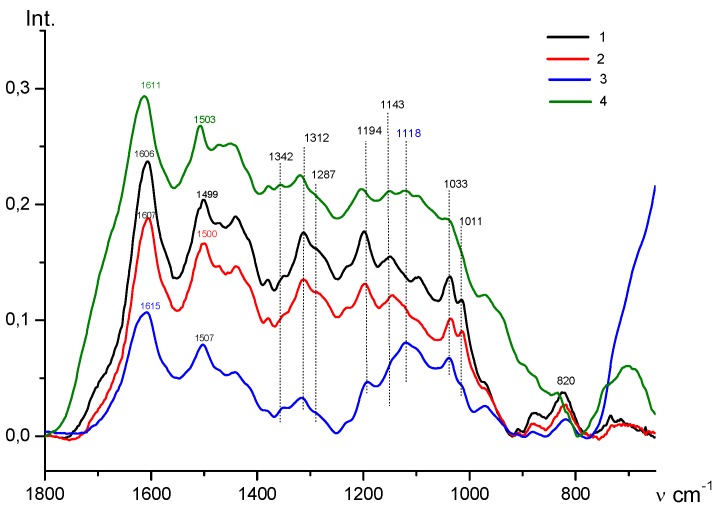
Attenuated total reflection (ATR) FTIR spectra of PAMMP (1) and Fe_3_O_4_/SWCNT/PAMMP nanocomposite, prepared at *C*_Fe_ = 17.6 (2,4) and 45.7% (3), and at *C*_SWCNT_ = 10 (2,3) and 20 wt % (4).

The FTIR spectroscopy data confirmed the immobilization of the monomer on the surface of the metal-carbon Fe_3_O_4_/SWCNT nanocomposite. The FTIR spectra of the Fe_3_O_4_/SWCNT/PAMMP nanomaterial (Figure 4) show a shift of absorption band at 556 to 572 cm^−1^, corresponding to stretching vibrations of the ν_Fe–O_ bond. In the absorption bands this shift indicates donor–acceptor interaction of PAMMP with Fe_3_O_4_ nanoparticles. An increase in the content of Fe_3_O_4_ in the Fe_3_O_4_/SWCNT/PAMMP nanocomposite leads to a significant growth in the intensity of the band at 572 cm^−1^. At the same time, a band appears at 1118 cm^−1^ in the ATR FTIR spectra of the nanocomposite (Figure 6), being an overtone of the main iron oxide band at 572 cm^−1^ (ν_Fe–O_).

All the main bands typical of the chemical structure of PAMMP remained in the FTIR spectra of the Fe_3_O_4_/SWCNT/PAMMP nanocomposite (Figure 4 and Figure 6) [25]. The intense bands at 1606 and 1499 cm^−1^ correspond to stretching vibrations of ν_C–C_ bonds in aromatic rings. Absorption bands at 1342, 1312, and 1226 cm^−1^ are related to stretching vibrations of ν_C–N_ bonds. Bands at 1194 and 1143 cm^−1^ are typical for in-plane bending vibrations of δ_C–H_ bonds of the aromatic ring [33,34,35,36,37]. Bands at 1033 and 1011 cm^−1^ are caused by out-of-plane bending vibrations of δ_C–H_ bonds of the aromatic ring. Bands at 731 and 714 cm^−1^ are related to out-of-plane bending vibrations of δ_C–H_ bonds in the trisubstituted benzene ring of the end groups.

The presence of absorption bands at 820 and 1287 cm^−1^ (out-of-plane bending vibrations of δ_C–H_ bonds in the 1,2,4,5-substituted benzene ring) indicates that the polymer chains grow via the C–N bonding between 3-amine groups and the para position of the phenyl rings in relation to the nitrogen. This type of bonding is observed in the course of aniline polymerization (“head-to-tail” type) [33,38,39].

A characteristic change in the FTIR spectra of the nanocomposite compared to the polymer spectrum is that the increase in content of carbon nanotubes results in a hypsochromic shift of frequency of skeleton vibrations of PAMMP by 7–9 cm^−1^ in the ATR FTIR spectra of the Fe_3_O_4_/SWCNT/PAMMP nanocomposite (Figure 6). In the absorption bands this shift indicates π–π* interaction of phenazine units of PAMMP with the aromatic structures of SWCNT.

**Figure 7:**
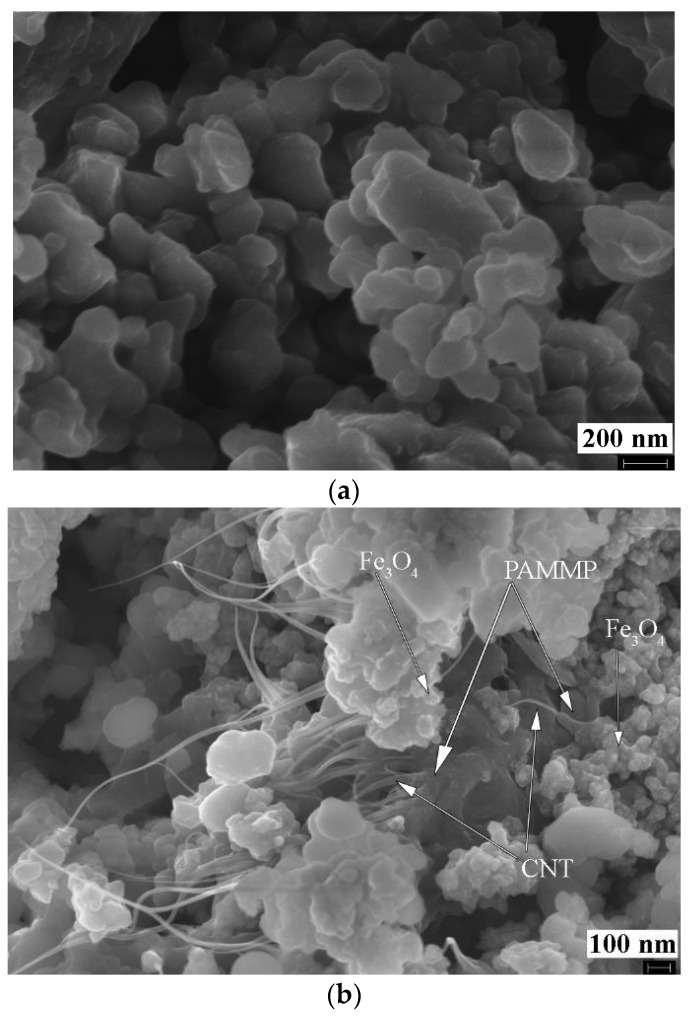
Field Emission (FE)-SEM images of the PAMMP (**a**) and Fe_3_O_4_/SWCNT/PAMMP nanocomposites (**b**).

**Figure 8:**
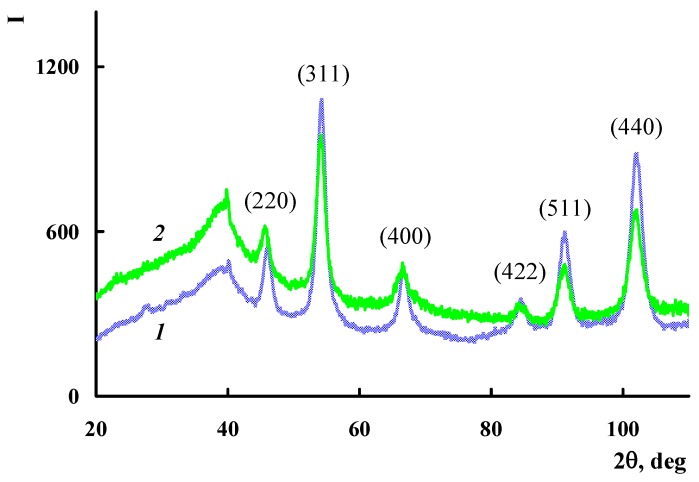
X-ray diffractograms of the Fe_3_O_4_/SWCNT/PAMMP nanocomposite, prepared at *C*_SWCNT_ = 10 (1) and 20 wt % (2).

In the process of in situ oxidative polymerization of ADMPC on the surface of Fe_3_O_4_/SWCNT nanocomposite, PAMMP phenazine units provide the formation of polymer chains in the immediate vicinity of the metal-carbon Fe_3_O_4_/SWCNT surface. According to TEM and FE-SEM data, PAMMP is formed on the surface of the Fe_3_O_4_/SWCNT nanocomposite (Figure 3 and Figure 7). This is also confirmed by BET surface area test data. The specific surface area of the Fe_3_O_4_/SWCNT/PAMMP nanocomposite (58.8 m^2^/g) is much smaller than that of SWCNT (1067.3 m^2^/g) and is closer to that of polymer (15.9 m^2^/g) (Table 2). According to the XRD data, the polymer component in the nanocomposite is amorphous (Figure 5 and Figure 8).

The diffraction patterns of the Fe_3_O_4_/SWCNT/PAMMP nanocomposite identify clearly the diffraction peaks of Fe_3_O_4_ at scattering angles 2θ = 46.1°, 54.2°, 66.9°, 84.8°, 91.2°, and 102.2° (Cr*K*_α_-radiation) (Figure 5 and Figure 8). These diffraction peaks refer to the Miller Indices (220), (311), (400), (422), (511), and (440) [32]. According to the TEM and FE-SEM data, the size of Fe_3_O_4_ nanoparticles determined using the EsiVision software is within the range of 2 < d < 8 nm (Figure 3 and Figure 7). According to AAS data, depending on the synthesis conditions, the content of *C*_Fe_ = 17.6–45.7% (Table 1).

**Figure 9:**
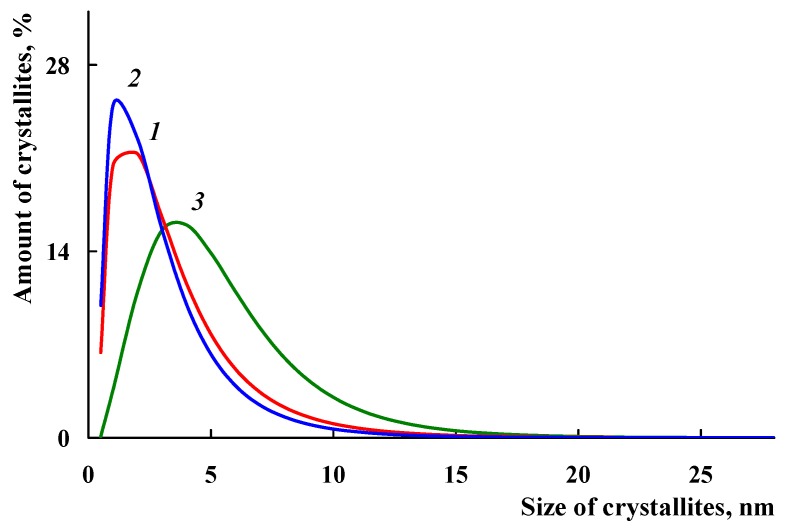
Fe_3_O_4_ crystallites size distribution in Fe_3_O_4_/SWCNT/PAMMP (1,2) and Fe_3_O_4_/SWCNT (3) nanocomposites, prepared at *C*_Fe_ = 17.6 (1) and 45.7% (2).

Figure 9 shows the size distribution of the coherent scattering regions in Fe_3_O_4_ nanoparticles. In the Fe_3_O_4_/SWCNT/PAMMP nanocomposite, the size distribution curves of Fe_3_O_4_ crystallites are narrow. About 95–97% of Fe_3_O_4_ crystallites have sizes up to 8 nm. As it can be seen in Figure 9, the Fe_3_O_4_/SWCNT nanocomposite has a wider size distribution curve. Only about 85% of Fe_3_O_4_ crystallites have sizes up to 8 nm. According to TEM data, the size of Fe_3_O_4_ nanoparticles in the Fe_3_O_4_/SWCNT nanocomposite is within the range of 6 < d < 15 nm (Figure 3a). This is explained by the fact that the polymer on the surface of Fe_3_O_4_/SWCNT reduces the aggregation of nanoparticles in the course of the synthesis of the Fe_3_O_4_/SWCNT/PAMMP nanomaterial.

**Figure 10:**
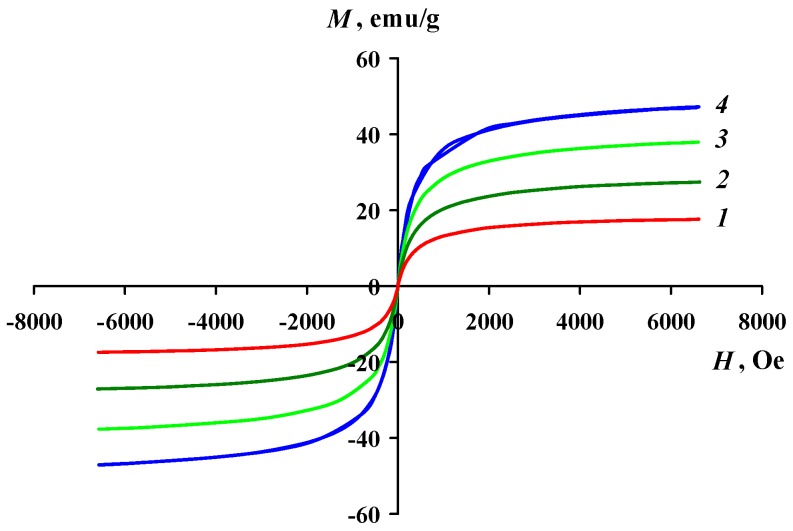
Magnetization of the Fe_3_O_4_/SWCNT/PAMMP nanocomposite as a function of applied magnetic field at room temperature, where Fe_3_O_4_/SWCNT/PAMMP was prepared at *C*_Fe_ = 17.6 (1), 26.6 (2), 42.4 (3), and 45.7% (4).

**Figure 11:**
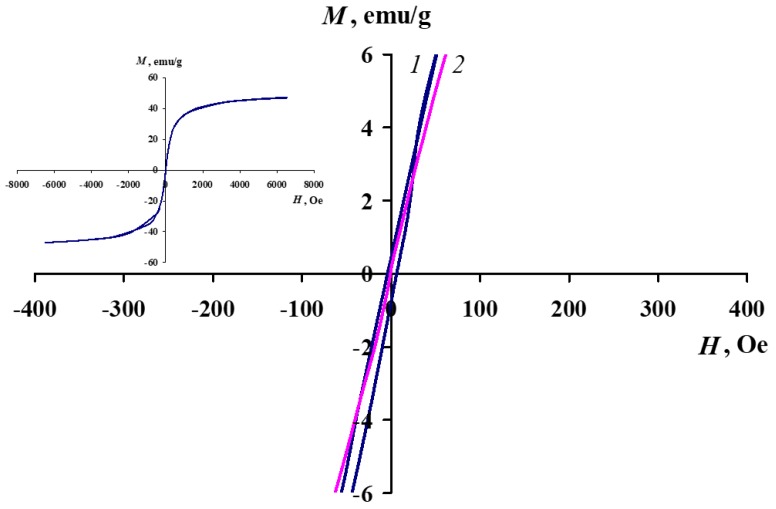
Magnetization of Fe_3_O_4_/SWCNT (1) and Fe_3_O_4_/SWCNT/PAMMP (2) nanocomposites as a function of applied magnetic field at room temperature.

The magnetic properties of Fe_3_O_4_/SWCNT and Fe_3_O_4_/SWCNT/PAMMP nanomaterials have been studied and the values of their main magnetic characteristics have been measured. The magnetization dependence on the magnetic field intensity at room temperature is shown in Figure 10 and Figure 11. The effect of iron concentration on the magnetic properties of the Fe_3_O_4_/SWCNT/PAMMP nanomaterial has been investigated. The saturation magnetization *M*_S_ depends on the iron concentration and reaches 47.24 emu/g at *C*_Fe_ = 45.7% (Table 1), which is significantly higher than those of other similar materials reported in previous studies [20,22]. The hysteresis loop squareness coefficient *κ*_S_ = *M*_R_/*M*_S_ ~ 0 indicates the superparamagnetic behavior of the hybrid nanomaterial [29,40]. As it can be seen in Figure 11, in the Fe_3_O_4_/SWCNT nanocomposite, the hysteresis loop squareness coefficient is slightly higher (*κ*_S_ = 0.01). The residual magnetization *M*_R_ of neat Fe_3_O_4_/SWCNT is 0.45 emu/g, and the coercive force *H*_C_ is 6 Oe. The superparamagnetic behavior of nanocomposites at relatively high concentrations of magnetic nanoparticles is specified by the small sizes and high dispersity of the magnetic nanoparticles. Anyway, the obtained values of *M*_R_/*M*_S_ for Fe_3_O_4_/SWCNT/PAMMP and Fe_3_O_4_/SWCNT nanocomposites are characteristic of uniaxial, single-domain particles. The critical size of the transition to a single-domain state for Fe_3_O_4_ is 128 nm [29,40].

**Figure 12:**
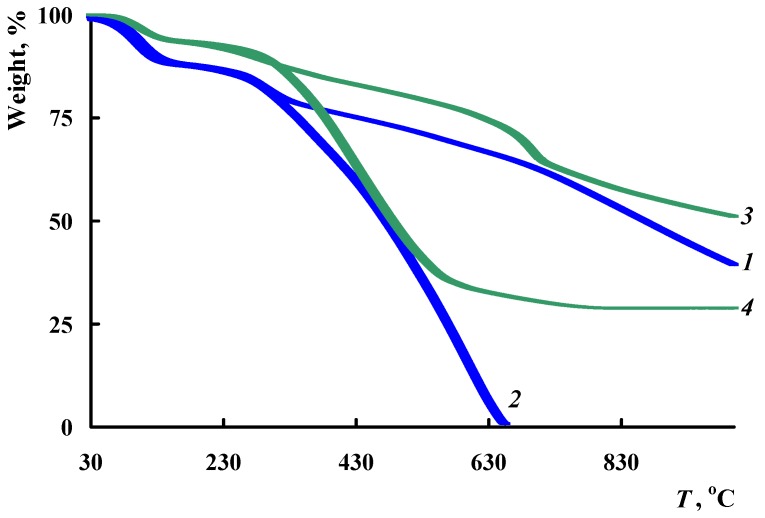
TGA thermograms of the PAMMP (1,2) and Fe_3_O_4_/SWCNT/PAMMP nanocomposites (3,4) at heating of up to 1000 °C in the Ar flow (1,3) and in air (2,4).

**Figure 13:**
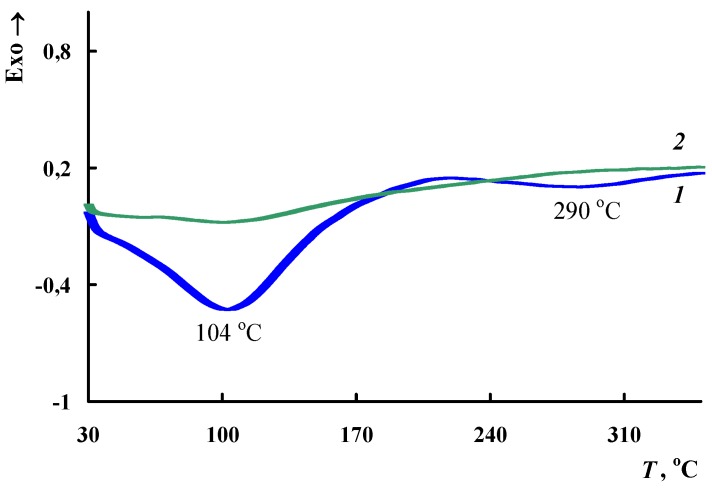
DSC thermograms of the Fe_3_O_4_/SWCNT/PAMMP nanocomposite upon heating in the nitrogen flow to 350 °C (1—first heating, 2—second heating).

TGA and DSC methods were used to study the thermal stability of the Fe_3_O_4_/SWCNT/PAMMP nanocomposite. Figure 12 shows the temperature dependence on weight loss in the Fe_3_O_4_/SWCNT/PAMMP nanocomposite compared to PAMMP when heated up to 1000 °C in the argon flow and in air. The nanocomposite content of carbon nanotubes *C*_SWCNT_ = 10 wt % relative to the monomer weight. According to AAS data, the content of *C*_Fe_3_O_4__ = 23.5%. In Table 3, the main thermal properties of obtained materials are listed. As it is seen in Figure 12, the character of the weight loss curves does not change until 320 °C. The weight loss at low temperatures is associated with the removal of moisture, which is confirmed by the DSC data (Figure 13).

The thermal stability of the Fe_3_O_4_/SWCNT/PAMMP nanocomposite is slightly higher than that of PAMMP. In an inert medium above 320 °C, the weight loss of the samples occurs gradually. PAMMP loses half of its initial weight in an inert atmosphere at 865 °C. For the nanocomposite at this temperature, the weight loss is only 44% and at 1000 °C the residue is 51%. The processes of thermo-oxidative degradation begin at 315 °C. The 50% loss of weight is observed at 475 °C for the polymer, and at 485 °C for the nanocomposite.

**Figure 14:**
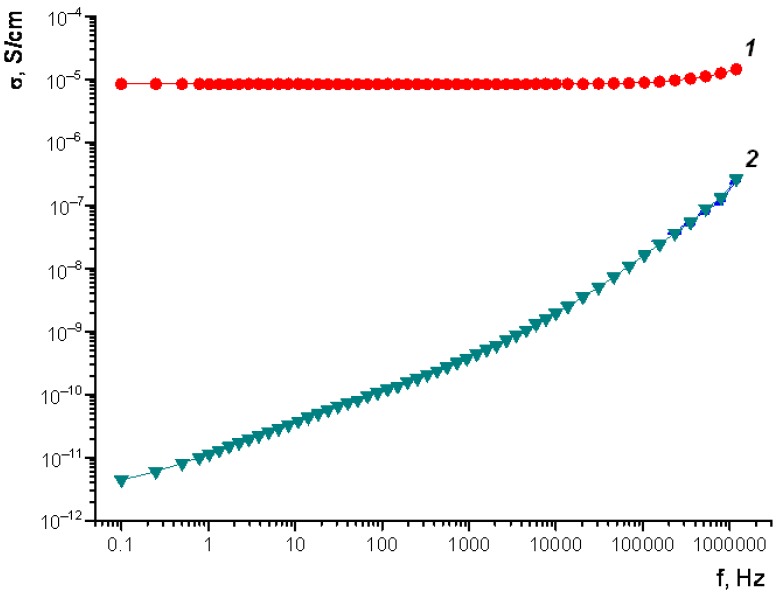
Frequency dependence on the conductivity for the Fe_3_O_4_/SWCNT/PAMMP (1) and SWCNT/PAMMP (2) nanocomposites, prepared at *C*_SWCNT_ = 3 (2) and 10 wt % (1) and at *C*_Fe_ = 17.6% (1).

The frequency dependence on the ac conductivity (σ_ac_) in the Fe_3_O_4_/SWCNT/PAMMP nanocomposite has been studied. The Fe_3_O_4_/SWCNT/PAMMP nanocomposite demonstrates a weak frequency dependence on σ_ac_ conductivity in the range of 0.1–5.0 × 10^4^ Hz. As the frequency of the alternating current grows in the range of 7.7 × 10^3^–1.15 × 10^6^ Hz, the conductivity increases from 8.3 × 10^−6^ S/cm to 2.4 × 10^−5^ S/cm (Figure 14).

Meanwhile, the frequency dependence on the ac conductivity (σ_ac_) in the SWCNT/PAMMP nanocomposite, containing no magnetite nanoparticles, demonstrates a gradual increase in electrical conductivity in the whole of the investigated frequency range (curve 2 in Figure 14). As the frequency increases, the electrical conductivity rises up to a value of 3.4 × 10^−7^ S/cm. According to previous studies [41,42], such character of the frequency dependence on conductivity indicates a hopping mechanism of charge transfer. Therefore, the presence of Fe_3_O_4_ nanoparticles changes the nature of the nanocomposite electrical conductivity.

According to previous studies [41,42], the frequency dependence on the ac conductivity in the metal-containing polymer composites is described by evaluation:

σ*_ac_* = σ*_dc_*_+_*Aω^p^*

where ***ω*** = 2πf is the angular frequency, ***A*** and ***p*** depend on the temperature and the volume fraction of the conducting metal-containing component.

At low frequencies, the main contribution to the conductivity is made by the component σ*_dc_*. In this case, its contribution is greater as the metal content increases [43]. The measurements of conductivity σ*_dc_* showed that the Fe_3_O_4_/SWCNT/PAMMP conductivity σ*_dc_* = 8.3 × 10^−6^ S/cm is 6 orders higher in magnitude than the conductivity of PAMMP in the basic form (σ*_dc_* = 1.2 × 10^−12^ S/cm) and SWCNT/PAMMP (σ*_dc_* = 4.4 × 10^−12^ S/cm). Hence, the magnetite nanoparticles make a significant contribution to the conductivity σ*_dc_* of the nanocomposite. An ionic component of the conductivity also plays an important role in the low-frequency region. Thus, for the Fe_3_O_4_/SWCNT/PAMMP nanocomposite, weak conductivity changes in the low-frequency range are determined by a growth of percolation degree provided by the high content of magnetite and SWCNT presence, which increases the probability of indirect electron tunneling.

As the frequency increases, the influence of the ionic component on electrical conductivity is leveled and the hopping conduction mechanism begins to play a crucial role. Therefore, in the high-frequency region, the conductivity σ*_ac_* of the Fe_3_O_4_/SWCNT/PAMMP nanocomposite starts to rise.

## 4. Conclusions

Polymer-metal-carbon hybrid nanomaterials based on thermostable electroactive poly-3-amine-7-methylamine-2-methylphenazine (PAMMP), single walled carbon nanotubes (SWCNT), and magnetite (Fe_3_O_4_) nanoparticles were synthesized for the first time. The obtained thermostable ternary nanomaterials are multifunctional and exhibit certain electrical and magnetic properties. Fe_3_O_4_/SWCNT/PAMMP nanocomposites are superparamagnetic (*κ_n_* = *M*_R_/*M*_S_ ~ 0). The size of the Fe_3_O_4_ nanoparticles is within the range of 2 < d < 8 nm. The saturation magnetization *M*_S_ depends on the iron concentration and is 17.65–47.24 emu/g at *C*_Fe_ = 17.6–45.7%. The Fe_3_O_4_/SWCNT/PAMMP nanocomposite demonstrates a weak frequency dependence on the σ_ac_ conductivity. As the ac frequency rises in the frequency range of 7.7 × 10^3^ Hz–1.15 × 10^6^ Hz, the conductivity increases from 8.3 × 10^−6^ to 2.4 × 10^−5^ S/cm. The nanocomposite loses half of its initial weight in air at 485 °C. In an inert atmosphere at 1000 °C the residue is 51%. The prepared multifunctional hybrid nanomaterials are very promising for modern technologies and can be employed for information storage systems and for creating sensors, electromagnetic shields, materials absorbing electromagnetic radiation in different wavelength ranges, and contrast agents for magnetic resonance tomography. High thermal stability of the obtained hybrid nanomaterials in the air and in an inert atmosphere provides a possibility to use them in high temperature processes.

## Figures and Tables

**Table 1:** Magnetic properties of Fe_3_O_4_/SWCNT/PAMMP nanocomposite *.

Fe, % **	*H*_C_, Oe	*M*_S_, emu/g	*M*_R_, emu/g	*M*_R_/*M*_S_
17.6	0	17.65	0	0
26.6	0	27.41	0	0
42.4	0	37.94	0	0
45.7	1.1	47.24	0.24	0.005

* *C*_SWCNT_ = 10 wt % relative to the monomer weight, ** according to AAS data. *H*_C_: coercive force, *M*_S_: saturation magnetization, *M*_R_: residual magnetization.

**Table 2:** BET surface area test data of materials.

Materials	Surface Area, m^2^/g	Pore Volume, cm^3^/g
PAMMP	15.9	0.022
Fe_3_O_4_/SWCNT/PAMMP *	58.8	0.149
Fe_3_O_4_	114.3	0.272
SWCNT	1067.3	1.073

* *C*_SWCNT_ = 10 wt % relative to the monomer weight, *C*_Fe_ = 45.7%.

**Table 3:** Thermal properties of the materials.

Property	PAMMP	Fe_3_O_4_/SWCNT/PAMMP
* *T*_5%_, °C	87/96	124/124
** *T*_50%_, °C	474/865	485/>1000
*** Residue, %	0/39	29/51

* *T*_5%_, ** *T*_50%_—5% and 50% weight losses (Air/Ar), *** residue at 1000 °C (Air/Ar). *C*_Fe3O4_ = 23.5%.
